# Scaling up cancer care for children without medical insurance in developing countries: The case of Mexico

**DOI:** 10.1002/pbc.24265

**Published:** 2013-02

**Authors:** Ricardo Pérez-Cuevas, Svetlana V Doubova, Marta Zapata-Tarres, Sergio Flores-Hernández, Lindsay Frazier, Carlos Rodríguez-Galindo, Gabriel Cortes-Gallo, Salomon Chertorivski-Woldenberg, Onofre Muñoz-Hernández

**Affiliations:** 1 Division of Social Protection and Health, Inter American Development BankMexico City, Mexico; 2 Epidemiology and Health Services Research Unit CMN Siglo XXI, Mexican Institute of Social SecurityMexico City, Mexico; 3 Oncology Department, Hospital Infantil de México Federico GomezMexico City, Mexico; 4 Center of Research on Population Health, National Institute of Public HealthCuernavaca, Morelos, Mexico; 5 Harvard Medical School and Dana-Farber Cancer InstituteBoston, Massachusetts; 6 Medical Insurance for the New Generation ProgramMexico City, Mexico; 7 Ministry of HealthMexico City, Mexico; 8 Research Division, Hospital Infantil de México Federico GómezMexico City, Mexico

**Keywords:** childhood cancer, developing countries, survival experience

## Abstract

**Background:**

In 2006, the Mexican government launched the Fund for Protection Against Catastrophic Expenditures (FPGC) to support financially healthcare of high cost illnesses. This study aimed at answering the question whether FPGC improved coverage for cancer care and to measure survival of FPGC affiliated children with cancer.

**Procedure:**

A retrospective cohort study (2006–2009) was conducted in 47 public hospitals. Information of children and adolescents with cancer was analyzed. The coverage was estimated in accordance with expected number of incident cases and those registered at FPGC. The survival was analyzed by using Kaplan–Meier survival curves and Cox proportional hazards regression modeling.

**Results:**

The study included 3,821 patients. From 2006 to 2009, coverage of new cancer cases increased from 3.3% to 55.3%. Principal diagnoses were acute lymphoblastic leukemia (ALL, 46.4%), central nervous system (CNS) tumors (8.2%), and acute myeloid leukemia (AML, 7.4%). The survival rates at 36 months were ALL (50%), AML (30.5%), Hodgkin lymphoma (74.5%), Non-Hodgkin lymphoma (40.1%), CNS tumors (32.8%), renal tumors (58.4%), bone tumors (33.4%), retinoblastoma (59.2%), and other solid tumors (52.6%). The 3-year overall survival rates varied among the regions; children between the east and south-southeast had the higher risks (hazard ratio 3.0; 95% CI: 2.3–3.9) and 2.4; 95% CI: 2.0–2.8) of death from disease when compared with those from the central region.

**Conclusion:**

FPGC has increased coverage of cancer cases. Survival rates were different throughout the country. It is necessary to evaluate the effectiveness of this policy to increase access and identify opportunities to reduce the differences in survival.

## INTRODUCTION

Effective pediatric cancer care programs must encompass early detection, and financial support for treatment 1 as well as improvement of pediatric cancer units through well-trained healthcare professionals, who contribute to the multidisciplinary team approach. In developing countries childhood cancer is an emerging challenge; mortality rates are not improving and the cost of care is increasing 2. It is anticipated that by 2030, the developing world will account for 70% of all childhood cancers 3, 4, and the cancer-related mortality rate will be five per 100,000 children. This figure approximates to the rate reported in developed countries during the 1980s 5, 6.

Progress in cancer survival rates of children is attributed to long-term investment in health care infrastructure, and the application of disease-specific and risk-adapted treatment protocols 7–10. In the USA, between 1990 and 2004 death rates declined by 1.3% per year for all neoplasms 11. In Europe, between 1983–1992 and 1993–1997, the 5-year survival increased from 65% to 75% for all childhood cancers 12. The World Health Organization analysis of childhood cancer deaths in America, Asia, and Oceania, 1970 through 2007 reported that the average annual percent change in mortality from all childhood cancers in Mexico was +0.8% for females and +1% for males, while in developed countries was −3% for both 13; in this country pediatric cancer is the second most common cause of death among children aged 1–14 years 14.

Mexico is investing to reach equitable pediatric cancer care and minimize differences in mortality rates according to the geographic area of residence. The poorest states have reported the highest mortality rates 15, 16. Also, children without social security have a 30% greater risk of delayed diagnosis of cancer when compared with those without social security 17, 18.

To mitigate the burden of pediatric cancer, since 2004, the Mexican government implemented the Fund for Protection against Catastrophic Expenditures (FPGC is its acronym in Spanish), as part of the System of Social Protection for Health commonly known as “*Seguro Popular*.” FPGC financially supports health care by pooling the risks of high cost and low-prevalence diseases such as cancer, neonatal intensive care and HIV/AIDS 19, 20 to those patients that are not affiliated with the social security.

Before the FPGC was initiated, financial support was inadequate for children with cancer without social security. Families would have to assume all costs of care, although some support could be obtained from non-governmental organizations.

In 2006, FPGC began funding the treatment of children with acute lymphoblastic leukemia (ALL); in 2008 it extended its benefits to all types of childhood cancer. The financial support varies according to the type of cancer, ranging from Mx$77,080 (US$5,930) for retinoblastoma to Mx$396,544 (US$30,272) for acute myeloid leukemia (AML).

By 2009, 47 hospitals of the Ministry of Health (MoH) were affiliated with FPGC. All hospitals that manage children with cancer must be certified to receive FPGC funds. This means that the hospital must have well-trained healthcare professionals, and the resources needed to provide cancer care. The MoH issues the clinical protocols for the treatments that children should receive 21. The protocols are evidence-based and developed by experts at the Children's Oncology Group. These protocols include laboratory tests for diagnosis and follow-up, medications, ambulatory, and hospital services. The rationale behind these rules is that cost is not the only barrier for access; the MoH included the normative dimension of appropriate capability of public hospitals to provide care to children with cancer.

The three objectives of the study were to investigate the progress of FPGC to increase coverage of new patients suffering from childhood cancer, to describe their sociodemographic, clinical characteristics, and health outcomes, and to analyze overall survival.

## METHODS

This retrospective cohort study was conducted with data of the period comprised since the onset of FPGC in 2006 to September 2009. The study population was children and adolescents with newly diagnosed cancer who were cared for at 47 MoH hospitals affiliated with FPGC. The scientific review board and the ethics committee of Hospital Infantil de Mexico Federico Gomez reviewed and approved the study protocol.

For the purposes of this study, coverage measures the extent to which the provider satisfies the potential need for specific services in a given community 22. To investigate the progress of coverage, the estimated number of eligible children and adolescents to receive care by the MoH was used 23. The country was divided into five geographical regions and its corresponding states: West (Aguascalientes, Colima, Guanajuato, Jalisco, Michoacán, and Nayarit) Northwest (Baja California, Baja California Sur, Chihuahua, Durango, Sinaloa, and Sonora), Central (Distrito Federal, Hidalgo, México, Morelos, Querétaro, and Tlaxcala), East (Coahuila, Nuevo León, San Luis Potosí, Tamaulipas, and Zacatecas), and Southeast (Campeche, Chiapas, Guerrero, Oaxaca, Puebla, Quintana Roo, Tabasco, Veracruz, and Yucatán). The expected number of patients for all types of cancer was estimated by multiplying the number of eligible children to receive care (children without social security and cared for by the MoH) by 121.5 per 1,000,000 children/years. This is the age-standardized incidence rate for all types of cancer for individuals aged 0–14 years reported in Mexico 24. No incidence rate for individuals 15–19 years was available for Mexican population. The resulting number was divided by 1,000,000. The amount of new cancer patients from FPGC registries was identified, and the proportion of cancer cases funded in relation to the expected number of cases was calculated. The proportions were estimated by considering the number of new cancer patients registered as the numerator and the expected number of cancer patients as the denominator.

To describe the sociodemographic and clinical characteristics and analyze the survival experience, the analysis comprised the registries of the claims made by affiliated hospitals to FPGC for reimbursement, and information from clinical charts. A group of previously trained nurses visited the hospitals and filled out the data collection instrument elaborated *ad hoc*.

The main variables were divided in four areas: sociodemographic, children clinical characteristics, abandonment, and survival. The sociodemographic characteristics included geographical area of residence, mother's schooling. The clinical characteristics of children were: sex, age at diagnosis, previous disease, cancer type, and risk group of cancer according to its prognosis and stage at diagnosis, relapse, and adverse events (AE).

The diagnosis grouping was established in accordance with the Third Edition of the International Classification of Childhood Cancer 25. Children with high-risk disease were those suffering from high-risk ALL (children aged <1 or >10 years, with hyperleukocytosis, primary central nervous system (CNS) infiltration, T-cell immunophenotype, Philadelphia chromosome, t(4;11)(q21;q23), t(8;14)(q24.1;q32), complex karyotype (more than four abnormalities) low hypodiploidy, near tetraploidy; del 17p; t(11q23) 26, 27, high-risk AML (M0, M1, M2, M4, M5, M6, M7) 28, high-risk Hodgkin and non-Hodgkin Lymphoma (stages III and IV) 29, high-risk CNS tumors such as astrocytoma y meduloblastoma (stages III and IV, and metastatic) 30, and stages III and IV solid tumors 28. To be able to classify solid tumors correctly, all hospitals had access to a computed axial tomographer, bone scintigraphy, bone marrow biopsy, and lumbar puncture as international standards recommend.

The AE classification was in accordance with the National Cancer Institute, which organizes AE according to the System Organ Class groupings and severity grades 31. AE were registered when a doctor diagnosed the patient and he/she was admitted. AE appeared after diagnosis, regardless of treatment provision. Only AE graded between 2 and 5 were recorded.

Abandonment was defined as patients that only had up to two visits registered in the clinical chart, and without further registries. Survival was measured from the time of initial diagnosis to the date of death from any cause or to the last date of follow-up or last contact.

### Statistical Analysis

Descriptive analysis was performed by geographical region. The proportions for all categorical variables were obtained. The survival analysis included the 2006–2009 period. The Kaplan–Meier (KM) method was used to estimate survival curves 32 and their 95% CI (95% CI). The analysis included hematological malignancies: ALL, AML, Hodgkin lymphoma, and non-Hodgkin lymphoma; and solid tumors: CNS, osteosarcoma, Wilms' tumor, retinoblastoma, and all other causes of solid tumors. Censored cases were those without outcome events before the end of the study. Patients who died, who abandoned treatment or were lost to follow-up were considered as an AE. The log-rank test was used to compare survival curves.

To determine the impact of the program on the survival experience in terms of geographical region, bivariate analyses were performed to check for possible confounders. For the multivariate analysis the Cox proportional hazard regression model was used 33. The analysis comprised three multivariate models: all types of cancer, hematologic (ALL), and solid malignancies (CNS). The multivariate analyses included adjustment for the following factors: sex, socioeconomic level (mother's schooling), previous disease, age of diagnosis, cancer type, relapse, risk group of cancer, and AE by grading scale. For the purpose of the multivariate analysis the referent categories were central region, age 1–4 years at diagnosis, mother's high school literacy, patients without AE or with AE grade 1, and lymphoma for cancer type. The variables relapse and sex were dichotomized. The multivariate analyses were performed using the backward procedure with elimination based on *P* > 0.05. Hazard ratios with 95% CI were calculated for all types of cancer, ALL, and CNS tumors. The partial likelihood ratio test served to compare the models.

Graphical examination of the KM survival curves, time-dependent covariates in the Cox proportional hazards model, and a global test of proportionality with the Schoenfeld and scaled Schoenfeld residuals assessed the Cox proportional hazards assumption for individual variables. The final model comprised the estimates of the covariates of type of cancer, age of diagnosis, mother's schooling, risk group of cancer, treatment relapse, AE, and sex. All tests were two-tailed and *P*-values <0.05 were considered significant. The fit of the model was evaluated using the Cox–Snell residuals. All statistical analyses were performed with STATA version 10.0.

## RESULTS

Data were collected from 47 hospitals, accounting for 4,065 patients. The final analysis comprised 3,821 patients with newly diagnosed cancer and 244 cases who were diagnosed before the program began and entered during relapse.

Table I demonstrates the progress of coverage of new cancer patients. The total number of eligible (without social security) patients to receive care ranged from 25.4 million in 2006 to 24.4 million in 2009. The majority of patients were in the central and south regions. The number of hospitals varied per region. The estimates of the expected number of cancer patients demonstrated that central and south-southeast regions had the highest figures (774 and 1,118 in 2009, respectively). The highest increase in coverage was in the northwest (2.2% in 2006 to 77.7% in 2008), the lowest in the south-southeast (1.6% in 2006 to 37.8% in 2008).

**Table I:** Progress of Coverage of the *Fund for Protection against Catastrophic Expenditures* of Children and Adolescents With Newly Diagnosed Cancer

Years	Regions of the country					Total
		West	Northwest	Central	East		South-southeast
Number of children and adolescents cared for by the MoH						
2006	5,660,571	2,263,271	6,572,116	1,350,582	9,563,964	25,410,504
2007	5,562,671	2,248,625	6,519,560	1,343,704	9,450,423	25,124,983
2008	5,448,459	2,215,172	6,427,133	1,327,794	9,320,806	24,739,364
2009	5,348,326	2,197,436	6,373,420	1,319,652	9,198,152	24,436,986
Number of hospitals affiliated with FPGC	12	9	11	4	11	47
Expected number of cases for all types of cancer						
2006	688	275	799	164	1,162	3,087
2007	676	273	792	163	1,148	3,053
2008	662	269	781	161	1,132	3,006
2009	650	267	774	160	1,118	2,969
Number of new cancer cases covered by the FPGC						
2006	33	6	33	10	19	101
2007	300	181	397	110	347	1,335
2008	378	209	540	107	428	1,662
2009^a^	163	111	236	45	168	723
Proportion of cancer cases that FPGC funded with respect to the expected number	%	%	%	%	%	%
2006	4.8	2.2	4.1	6.1	1.6	3.3
2007	44.4	66.3	50.1	67.5	30.2	43.7
2008	57.1	77.7	69.1	66.5	37.8	55.3
2009^a^	50.1	83.4	60.9	56.2	30.0	48.7

a aInformation from the cancer cases who were registered from January to September 2009; the study was conducted from October to December 2009. FPGC: Fund for Protection against Catastrophic Expenditures; MoH: Ministry of Health.

Table II depicts the main characteristics of the population. In each of the regions, there was a higher proportion of males (55.6%). Illiterate mothers and those with primary schooling accounted for 40% of the sample. The percentages of children with previous diseases ranged from 1.5% (west) to 5.0% (south); the group of patients aged 1–4 years had the highest percentage (35.1%), followed by the group aged 5–9 years (26.5%). ALL was the most frequent diagnosis (46.4%), followed by CNS tumors (8.2%), and AML (7.4%); 58.6% was considered high-risk. 44.9% had AE; 30.9% had AE grades 2 and 3, and 13.8% had AE grades 4 and 5. The most frequent AE were infections and infestations (18.9%), followed by blood and lymphatic system disorders (18%). The east region had less AE documented than the other regions. At the time of data collection, 68.1% of patients were alive, 23.7% had died, 6.7% had abandoned treatment and 2% had missing data. The east region showed the highest proportion of abandonment.

**Table II:** Characteristics of Children With Cancer Covered by the *Fund for Protection Against Catastrophic Expenditures*

	West (n = 874) (%)	Northwest (n = 507) (%)	Central (n = 1,206) (%)	East (n = 272) (%)	South-southeast (n = 962) (%)	Total (n = 3,821), n (%)
Female sex	45.4	41.0	43.4	48.2	45.4	1,698 (44.4)
Mother schooling*						
Illiterate	6.6	8.3	6.0	2.9	13.5	310 (8.1)
Primary school	35.8	18.5	32.7	19.9	40.0	1,240 (32.5)
Secondary school	20.4	16.0	30.4	31.6	17.3	878 (23.0)
Preparatory school	7.1	9.7	13.5	10.3	6.8	367 (9.6)
High school	2.5	4.5	3.8	6.3	1.9	126 (3.3)
Missing date	27.6	43.0	13.6	29.0	20.6	900 (23.6)
Previous disease*	1.5	3.0	2.3	2.2	5.0	110 (2.9)
Age of diagnosis*						
<1 year	6.1	7.7	7.5	4.0	4.5	237 (6.2)
1–4 years	32.8	33.5	38.1	34.2	34.3	1,340 (35.1)
5–9 years	28.5	23.5	25.0	24.6	28.8	1,014 (26.5)
10–14 years	22.4	23.3	21.7	20.6	23.0	853 (22.3)
15–19 years	10.2	12.0	7.5	16.5	9.5	377 (9.9)
Type of cancer*						
Acute lymphoblastic leukemia	48.3	41.8	46.2	36.4	50.3	1,774 (46.4)
Acute myeloid leukemia	8.9	7.1	6.9	4.4	7.8	284 (7.4)
Hodgkin lymphoma	8.7	6.3	5.4	4.4	7.0	252 (6.6)
Non-Hodgkin lymphoma	5.1	4.3	4.1	6.3	4.8	179 (4.7)
Malignant bone tumors	4.9	4.7	4.6	11.0	6.5	216 (5.7)
Central nervous system tumors	8.5	11.8	7.7	9.9	6.2	314 (8.2)
Renal tumors	3.3	6.1	4.2	5.1	4.2	165 (4.3)
Retinoblastoma	2.6	1.8	7.0	4.8	2.8	157 (4.1)
Other	9.6	16	13.8	17.6	10.4	480 (12.6)
High-risk*	66.0	58.2	57.5	47.8	56.5	2,239 (58.6)
	West (n = 866)	Northwest (n = 500)	Central (n = 1,204)	East (n = 272)	South-southeast (n = 962)	Total (n = 3,804)
Adverse events (AE)*	45.1	40.2	52.5	16.1	45.7	1,707 (44.9)
AE by grading scale (indicators of severity)						
Adverse events grade 2 and 3*	30.7	33.9	31.8	13.2	33.5	1,181 (30.9)
Adverse events grade 4 and 5*	14.1	5.7	20.6	2.9	12.2	526 (13.8)
AE by system organ class groupings						
Infections and infestations*	17.5	21.5	22.6	7.0	17.5	721 (18.9)
Blood and lymphatic system disorders*	14.2	8.1	28.7	2.6	17.3	684 (18.0)
Gastrointestinal disorders*	9.3	2.4	11.4	1.5	9.1	323 (8.5)
Nervous system disorders*	2.3	2.0	2.7	1.5	4.8	113 (3.0)
Respiratory, thoracic and mediastinal disorders*	2.6	1.0	3.2	1.5	2.9	99 (2.6)
Metabolism and nutrition disorders*	1.7	1.0	3.6	0.0	2.9	91 (2.4)
Renal and urinary disorders	1.5	0.6	1.2	0.4	2.1	51 (1.3)
Others*	10.4	11.6	9.0	3.7	11.4	379 (9.9)
	West (n = 874)	Northwest (n = 507)	Central (n = 1,206)	East (n = 272)	South-southeast (n = 962)	Total (n = 3,821)
Health status*						
Alive	72.9	67.9	73.4	62.1	58.8	2,601 (68.1)
Dead	22.2	25.6	19.9	17.3	30.7	906 (23.7)
Treatment abandonment	2.1	4.1	4.4	20.2	9.3	237 (6.2)
Missing date	2.9	2.4	2.3	0.4	1.2	78 (2.0)

n, number of children; statistical analysis comprised comparisons among regions, *P*-value was calculated using χ^2^-test.

* *P* < 0.05. Previous disease included congenital anomalies or hereditary diseases (n = 58), anemia (n = 43), chronic infectious diseases like tuberculosis and HIV (n = 9).

**Fig 1:**
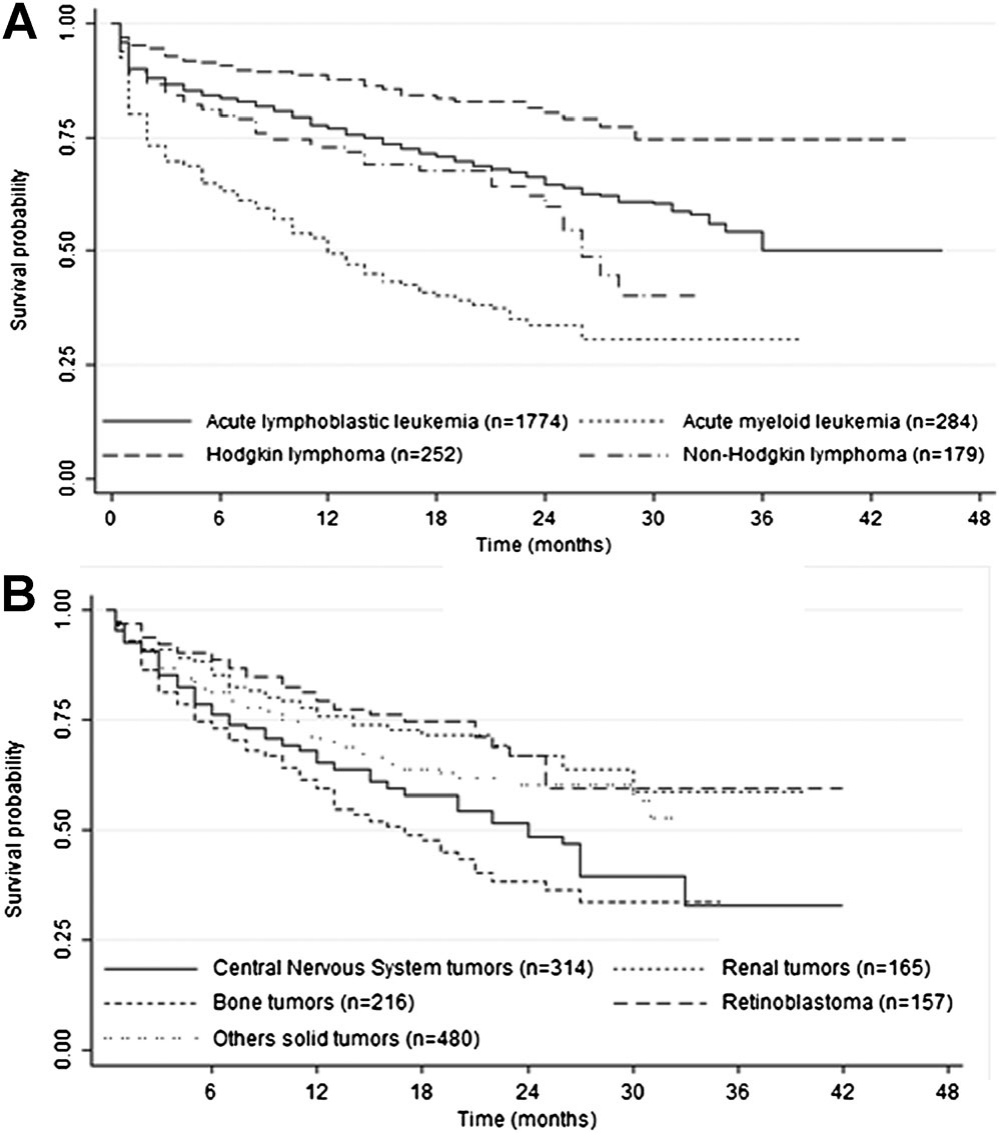
Estimates of survival for children financed by the Fund for Protection against Catastrophic Expenditures by type of cancer. A: Children with hematologic malignancies (n = 2,489). The 3-year survival rates (KM and 95% CI) were: ALL 50% (40.3–59), AML 30.5% (22.8–38.5), Hodgkin lymphoma 74.5% (64.5–82.1), and non-Hodgkin lymphoma 40.1% (25.1–54.6). Log-rank test, *P* < 0.0001. B: Children with solid tumors (n = 1,332). The 3-year survival rates (KM and 95% CI) were: CNS tumors 32.8% (19.2–47.1), Renal tumors 58.4% (43.0–70.9), Bone tumors 33.4% (23.4–43.7), Retinoblastoma 59.2% (46.1–70.1), and others solid tumors 52.6% (40.7–63.1). Log-rank test, *P* < 0.0001.

Figure 1A depicts the unadjusted KM survival curves of cases with hematologic malignancies. The survival estimates considered up to 36 months of follow-up of new cases. The survival rates were as follows: ALL 50% (95% CI: 40.3–59), AML 30.5% (95% CI: 22.8–38.5), Hodgkin lymphoma 74.5% (95% CI: 64.5–82.1), and non-Hodgkin lymphoma 40.1% (95% CI: 25.1–54.6). The log-rank test was *P* < 0.0001.

Figure 1B presents the unadjusted KM survival curves for children with solid tumors. The survival estimates comprised 36 months after diagnosis: the survival rates of CNS tumors were 32.8% (95% CI: 19.2–47.1); renal tumors: 58.4% (95% CI: 43.0–70.9), bone tumors: 33.4% (95% CI: 23.4–43.7); for retinoblastoma: 59.2% (95% CI: 46.1–70.1), and for other solid tumors: 52.6% (95% CI: 40.7–63.1). The log-rank test was *P* < 0.0001.

Figure 2A displays the estimated survival function of CNS tumors by region. The unadjusted KM survival curves for the five regions demonstrated that patients from the northwest and south-southeast had lower unadjusted survival estimates than the other regions. The 3-year survival rates by region were: west 53.8% (95% CI: 33.9–70.1), east 52.3% (95% CI: 28.7–71.5), center 43.9% (95% CI: 23.2–62.9), northwest 22.1% (95% CI: 4.9–47.0), and south-southeast 8.4% (95% CI: 0.7–29.5). The log-rank test was *P* < 0.005.

**2:**
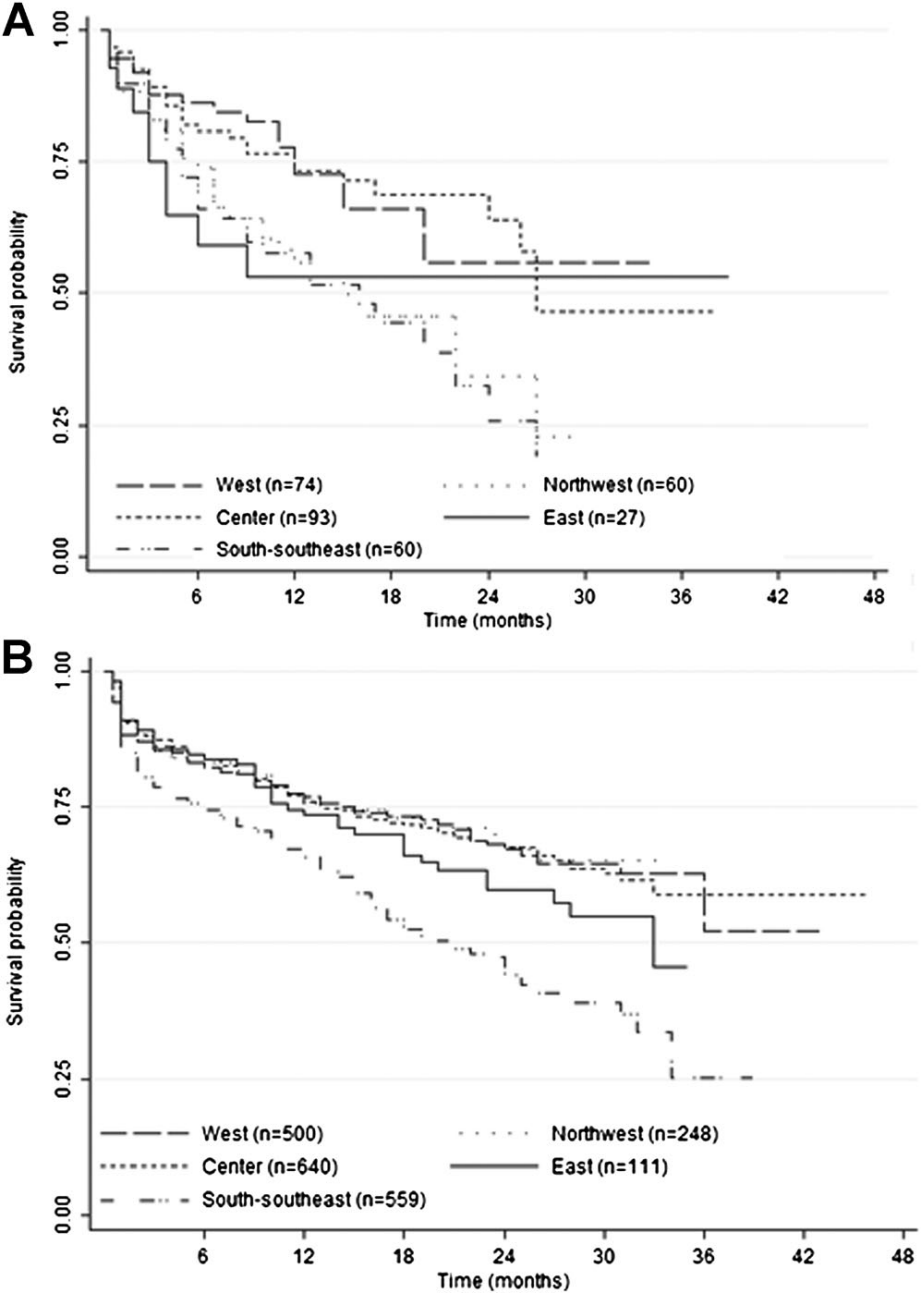
Estimates of survival for children financed by the Fund for Protection against Catastrophic Expenditures by geographic region. A: CNS tumors by region (n = 314). The 3-year survival rates (KM and 95% CI) were: west 53.8% (33.9–70.1), east 52.3% (28.7–71.5), center 43.9% (23.2–62.9), northwest 22.1% (4.9–47.0), and south-southeast 8.4% (0.7–29.5). Log-rank test, *P* < 0.005. B: Leukemia (ALL and AML, n = 2,048). The 3-year survival rates (KM and 95% CI) were: west 51.4% (31.1–68.4), northwest 64.6% (55.6–72.2), east 57.7% (49.4–65.1), center 43.4% (22.3–62.8), and south-southeast 21.3% (6.8–41.0). Log-rank test was *P* < 0.0001.

Figure 2B concentrates on the estimated survival function of leukemia (ALL and AML) by region. The plots of the unadjusted KM survival curves for the five regions are depicted in the same graph. The KM curve for the south-southeast was consistently lower than KM curves for other regions. After 12 months, as the number of follow-up months increased, the KM curves grew farther apart. The 3-year survival rates calculated in terms of geographical region were: west 51.4% (95% CI: 31.1–68.4), northwest 64.6% (95% CI: 55.6–72.2), east 57.7% (95% CI:49.4–65.1), center 43.4% (95% CI: 22.3–62.8), and south-southeast 21.3% (95% CI: 6.8–41.0). This indicates that children with cancer in the south-southeast region had poorer survival prognosis than children from the other regions. The log-rank test was *P* < 0.0001.

Table III summarizes the Cox proportional hazards model analyzing the influence of the region and other covariates on the survival experience of children with cancer in three groups: all types of cancer, ALL, and CNS tumors malignancies. The multivariate model including “all types of cancer” showed that children from all regions when compared with the central region had a significant risk of death; the east and south-southeast regions had the highest risk (hazard ratio 3.0; 95% CI: 2.3–3.9; 2.4, 95% CI: 2.0–2.8, respectively). Children whose age at diagnosis was less than 1 year or 15–19 years of age, those with illiterate mothers, at high risk, had relapse, or had AE grades 2–3 and 4–5, were at higher risk of death. Analysis of diagnosis demonstrated that patients with CNS tumors and bone tumors were at higher risk of death than individuals diagnosed with other cancers. The multivariate analysis for leukemia demonstrated similar risks; although, females with hematological malignancies had lower probability of death than boys (HR 0.9; 95% CI: 0.7–1.0, *P* = 0.055). The analysis of CNS tumors showed higher risk of death in children in the east region (HR 2.6; 95% CI: 1.0–6.2), northwest region (HR 2.5; 95% CI: 1.3–5.0), and the south-southeast (HR 2.2; 95% CI: 1.2–4.1).

**Table III:** Influence of Geographical Region and Other Covariates on Survival of Children With Cancer Covered by the *Fund for Protection Against Catastrophic Expenditures*

	All types of cancer			Leukemia					CNS tumors						
	Hazard ratio	95% CI	Hazard ratio	95% CI	Hazard ratio	95% CI
Geographical region						
West	**1.4**	**1.1**–**1.7**	**1.4**	**1.1**–**1.8**	1.1	0.5–2.5
Northwest	**1.8**	**1.4**–**2.4**	**1.5**	**1.0**–**2.3**	**2.5**	**1.3**–**5.0**
East	**3.0**	**2.3**–**3.9**	**2.7**	**1.8**–**4.1**	**2.6**	**1.0**–**6.2**
South-southeast	**2.4**	**2.0**–**2.8**	**2.6**	**2.0**–**3.2**	**2.2**	**1.2**–**4.1**
Age of diagnosis						
<1 year	**1.7**	**1.2**–**2.2**	**2.2**	**1.5**–**3.2**	1.1	0.3–3.3
5–9 years	1.1	0.9–1.3	1.2	0.9–1.6	0.9	0.5–1.6
10–14 years	1.2	0.9–1.5	**1.4**	**1.1**–**1.8**	0.6	0.3–1.3
15–19 years	**1.5**	**1.2**–**1.9**	**1.8**	**1.3**–**2.6**	1.9	0.6–6.2
Mother schooling						
Illiterate	**1.7**	**1.1**–**2.6**	**1.8**	**1.0**–**3.2**	1.5	0.4–4.5
Primary school	1.3	0.9–1.9	1.4	0.8–2.4	1.4	0.5–3.6
Secondary school	1.2	0.8–1.8	1.3	0.7–2.3	1.2	0.4–3.2
Preparatory school	1.1	0.8–1.7	1.3	0.7–2.4	1.2	0.4–3.8
High risk	**1.3**	**1.2**–**1.6**	**1.4**	**1.1**–**1.8**	0.9	0.4–1.4
Treatment relapse	**1.2**	**1.0**–**1.4**	**1.3**	**1.0**–**1.7**	1.1	0.5–2.2
Adverse events						
Grades 2–3	**1.4**	**1.2**–**1.7**	**1.4**	**1.1**–**1.8**	0.9	0.6–1.5
Grades 4–5	**4.9**	**4.1**–**5.9**	**5.1**	**4.0**–**6.5**	2.3	0.9–5.9
Female sex	0.9	0.8–1.0	0.9	0.7–1.0	1.3	0.8–2.1
Type of cancer						
Leukemia	1.1	0.8–1.4				
CNS tumors	**2.0**	**1.4**–**2.7**				
Retinoblastoma	1.4	0.9–2.3				
Renal tumors	1.3	0.8–2.0				
Malignant bone tumors	**1.8**	**1.3**–**2.5**				
Others	1.3	0.9–1.7				

Bold values indicate statistically significant results (*P* < 0.05) in the Cox proportional hazards model.

## DISCUSSION

This study demonstrates the progress of FPGC in increasing coverage for cancer care for children and adolescents that receive financial support and a wide variability in survival experiences of the new cases funded by this program across the regions in the country. After 4 years approximately 50% of expected number of pediatric cancer cases (without social security) had being funded to receive healthcare. This demonstrates increase in FPGC coverage, but our data do not support if there was an increase in supply for cancer care (i.e., the number of pediatric cancer units). Access for cancer care does not rely only on monetary resources, it also requires availability of appropriate services 34; yet, is reasonable to assume that hospitals increased their capability to be certified and receive FPGC funds. Furthermore, the actual number of children treated may not have increased, although the cost of care shifted from the parents or other funders to FPGC, thus reducing the number of out-of-pocket payers.

The percentage of abandonment of treatment was lower than reported in other studies. In Latin American countries, abandonment rates range from 10% to 24%, even in cases where treatment was financially covered 35. Various factors are associated with abandonment. Families with limited resources had lower adherence to treatment and increased delay of appointments and to buy medications 36. The overall abandonment rate in this study was 6.2%, although in the east region it reached 20.2%. Further studies would be beneficial to understand better the problem and develop interventions to improve adherence.

The prevalence of the various types of childhood cancer in this study was similar to the pattern in Latin America where acute leukemia is the most prevalent, followed by lymphomas and CNS tumors 37. Nonetheless, these results should be taken with caution and not be interpreted as representative of the incidence of cancer in this population. The proportional number of ALL was probably related to the fact that this was the first condition that FPGC financed and the other types of cancer were included gradually to the program. The condition of the patient at diagnosis was also noteworthy; 51% of patients were at high risk when diagnosed. These data suggest that these children do not have access to diagnostic services at early stages of the condition 38. The survival rates of Mexican children were sub-optimal when compared with developed countries. Overall survival analysis should include patients with a 5-year follow up period. However, the analysis was carried out with a 3-year follow up period, as this is the length of time that the fund to care for children with cancer has been in place. The survival rate for ALL was 50% at 36 months follow up. This group included standard and high-risk leukemia. In the United States, the Surveillance Epidemiology and End Results (SEER) 39 data report 5-year survival of 77% and St Jude Children's Research Hospital data reports 5-year survival data >90% 40. This report includes patients with high-risk ALL from 1983 with a 73% prediction of cure 41. The survival rate of AML was 30.5%. The current treatment results in a 60% survival rate for AML patients 42.

Regarding Hodgkin lymphoma, the survival rate was 74.5% at 36 months. SEER reported a 96% 5-year survival in adolescents 43, 44. Non-Hodgkin lymphoma had a survival rate of 40.1% at 36 months. It is difficult to make comparisons with this type of malignancy as various treatments are available, and a minimum of three histological types exists: Burkitt, anaplastic, and lymphoblastic lymphomas. However, a report that included all histological types estimated a 76.2% survival rate in adolescence and an 81% survival rate in childhood 45.

Survival time varied among the regions. The east and south-southeast regions had the poorest outcomes. Several aspects may influence the differences observed in survival rates among regions. The policy of funding all childhood cancer treatments is relatively new and entered gradually in the country. Therefore, it will require more time and additional resources to achieve reasonable outcomes. Further investigation and follow-up is required. For example, analyzing the health production function and survival rates of individual hospitals or among children with and without social security should help to determine benchmarks within the Mexican health sector.

A weakness of the study is the use of the age-standardized incidence rate for all types of cancer for cases aged 0–14 to estimate the number of expected cases for all types of cancer for children aged 0–19. Since the inception of the study we searched for the age-standardized incidence rate for children 0–14 and for adolescents 15–19 in Mexico; however, only the figure for the age group 0–14 years was available. In the US the incidence rates for 0–14 and for adolescents are 151 and 210 per million, respectively 46. These rates appear to be higher than the ones reported for Mexican children; if such figures were used, the incidence rate for the 15–19 years group would be overestimated. Though the incidence rate is higher among adolescents, we did not have an educated guess for Mexican population; therefore, we decided to use the same figure, although, the need exist to have these figures and the potential effect of underestimating the coverage for the group of adolescents.

This study analyzed the period during which funds for cancer for children affiliated with FPGC have been in existence. In-depth analysis of the adherence to treatment protocols was not carried out as all MoH hospitals had standardized therapeutic protocols. However, severe AE (13.8%) were frequent and this requires additional analysis. It is expected that patients suffer severe AE; however, if they receive inadequate treatment, this can be the cause of death.

From 2006 to 2009, FPGC has increased coverage of cancer cases that receive financial support from 3.3% to 55.3%. However, survival rates were different throughout the country. Using this report as a baseline, it will be imperative to continue to evaluate the effectiveness of this policy to increase access and identify opportunities to reduce the regional disparities in survival. The findings could be used to build on the knowledge derived from low and middle-income country's experiences concerning the advancement of policies to improve cancer care for children without medical insurance.

## Notes

Authors' contributions: Ricardo Pérez-Cuevas conceptualized and designed the study, coordinated the fieldwork, wrote the paper, and did the manuscript final version approval. Svetlana Doubova did the literature review, reviewed the database, did statistical analysis and interpretation of the data, and contributed in drafting the paper. Marta Zapata-Tarres contributed in the design of the study, developed the data collection instruments, did the field staff training, and revised critically the paper. Sergio Flores-Hernández did the statistical analysis and interpretation of the results. Lindsay Frazier reviewed the results of the study and revised critically the paper for important intellectual content. Carlos Rodriguez-Galindo reviewed the results of the study and revised the paper critically for important intellectual content. Gabriel Cortes-Gallo participated in the conception of the study, reviewed the results, and did the manuscript final approval. Salomon Chertorivski-Woldenberg participated in the conception of the study and did the manuscript final approval. Onofre Muñoz-Hernández participated in the conception and design of the study, and did the manuscript final approval.
